# We Run This City: Impact of a Community–School Fitness Program on Obesity, Health, and Fitness

**DOI:** 10.5888/pcd15.160471

**Published:** 2018-05-03

**Authors:** Elaine A. Borawski, Sarah Drewes Jones, Laura Danosky Yoder, Tara Taylor, Barbara A. Clint, Meredith A. Goodwin, Erika S. Trapl

**Affiliations:** 1 Prevention Research Center for Healthy Neighborhoods and Department of Population and Quantitative Health Sciences, Case Western Reserve University, Cleveland, Ohio; 2 Department of Nutrition, Case Western Reserve University, Cleveland, Ohio; 3 Prevention Research Center for Healthy Neighborhoods, Case Western Reserve University, Cleveland, Ohio; 4 YMCA of Greater Cleveland, Cleveland, Ohio

## Abstract

**Introduction:**

The We Run This City (WRTC) Youth Marathon Program is a community-supported, school-based fitness program designed to increase physical activity in a large, urban school district by engaging middle school youth to train 12 to 14 weeks to run or walk 1.2 miles, 6.2 miles, or 13.1 miles of the Rite Aid Cleveland Marathon. The objective of our study was to evaluate the effect of the intervention on adolescent health.

**Methods:**

We assessed changes in obesity, health, and fitness, measured before training and postintervention, among 1,419 sixth- to eighth-grade students participating in WRTC for the first time, with particular interest in the program’s effect on overweight (85th–94th body mass index percentile) or obese (≥95th percentile) students. We collected data from 2009 through 2012, and analyzed it in 2016 and 2017. Outcomes of interest were body mass index (BMI), waist-to-hip ratio (WHR), elevated blood pressure, and fitness levels evaluated by using the Progressive Aerobic Cardiovascular Endurance Run (PACER) test and the sit-to-stand test.

**Results:**

We saw significant improvements overall in fitness and blood pressure. Controlling for demographics, program event, and training dosage, BMI percentile increased among normal weight participants and decreased among overweight and obese participants (*P* < .001). WHR increased among obese participants, whereas reductions in blood pressure among those with elevated blood pressure were associated with higher amounts of training and lower baseline BMI.

**Conclusion:**

Even small amounts of regular physical activity can affect the health and fitness of urban youths. School–community partnerships offer a promising approach to increasing physical activity by supporting schools and making a school-based activity inclusive, fun, and connected to the broader fitness community.

## Introduction

More than one third of adolescents in the United States are overweight or obese, and rates of obesity are significantly higher among racial/ethnic minority youths and in economically disadvantaged communities (1–3). Decreasing levels of physical activity in the school day, combined with living in communities where resources are limited or built environments are not conducive to unsupervised outdoor play, is often identified as a contributing factor to the obesity epidemic among children and adolescents (3–6). Yet, schools are also frequently viewed as a prime venue for interventions aimed at reversing the trends in physical inactivity and obesity (7,8), with growing evidence of the effectiveness and sustainability of school-based interventions (9–14). In response to these limitations and opportunities, some researchers have suggested that the most effective interventions for increasing physical activity among adolescents are conducted not only in schools but also involve the community and families (15). Such interventions require a unique set of partnerships, and we evaluate the impact of one such program, the We Run This City (WRTC) Youth Marathon Program.

The WRTC program is a school-based youth fitness program in Cleveland, Ohio, developed by a multiorganization collaborative led by the YMCA of Greater Cleveland. It is implemented across a large metropolitan school district where most (85%) students are from racial/ethnic minority groups and from low-income households. The program was designed to address the declining levels of school-based physical education across the school district, where only 20% of middle school students were receiving daily physical education and more than 40% of middle school youth were considered overweight or obese (16).

The WRTC program encourages middle school youths (grades 6–8) at varying fitness levels to adopt active, healthy lifestyles and to engage in a goal-oriented activity: to train in a graduated manner over 12 to 14 weeks to run or walk a segment of 1.2 miles, 6.2 miles (10K), or 13.1 miles (half marathon) of the Rite Aid Cleveland Marathon. We examined the effect of the WRTC program on body mass index (BMI) and other body measurements, on blood pressure, and on overall fitness. A secondary aim was to examine the program’s impact on students who were overweight or obese before enrolling in the program.

## Methods

### Study design

We used a nonexperimental pretest­–posttest study design to examine the effect of the WRTC program by aggregating WRTC program evaluation data collected from 2009 through 2012, which we analyzed in 2016 and 2017. No reference group was available. Data were derived from all first-time participants enrolled in the program from 2009 through 2012 (n = 1,419), representing 60.4% of all WRTC participants and involving 35 schools. These aggregated data consisted of samples of 387 students in 2009, 407 in 2010, 452 in 2011, and 173 in 2012. Annual participation in the program at the school level varied, with 57% of schools participating for 1 to 2 years and 43% participating for 3 to 4 years. However, we included only first-time participants on each team in our analyses. Team sizes ranged from 2 to 72 students. Parental consent and student assent for study and program participation were obtained. The institutional review board of Case Western Reserve University approved the study.

### Intervention program

WRTC recruitment began in the fall of each year. Teams, typically led by a physical education teacher, were formed within schools and were composed of students with a range of fitness levels, athletic ability, and body composition. Other school personnel (classroom teachers, food service workers, security guards) also led or assisted with teams. Coaches were responsible for scheduling and leading practices, collecting training data, and ensuring that their teams attended prerace events (ie, evaluation, conditioning clinics, and practice races). Training for the 10K or the half marathon event (referred to hereinafter as 10K/half) began in January; training for the 1.2-mile event started mid-February. Race day is always the third Sunday of May.

The goal of training was to help participants safely build up to running their chosen event, allowing each student to progress at his or her own pace. Because of safety concerns (eg, crime, cracked or uneven sidewalks, dogs), most teams trained on school property either by running several laps around the gymnasium, creating a short course throughout school hallways, or running the perimeter of school grounds outdoors. Typically, trainings focused on endurance (continuous running or walking for a period of time) rather than distance.

Coaches were given a training curriculum, and trainings were held a minimum of 2 to 3 days per week depending on the event: 1.2-mile event participants trained twice per week and 10K/half participants trained at least 3 times per week. For both groups, the goal was to reach at least 25 miles of training before race day; however, the 10k/half participants were expected to log in at least 3 times that amount of training when including the required out-of-school practice races. Training focused on walking and running but also included sessions on nutrition (eg, intake of sugar-sweetened beverages or water), injury prevention, getting adequate sleep, and game-like activities (eg, relay races, obstacle courses). Training materials were developed by the YMCA and were drawn from national fitness initiatives such as Let’s Move (17). Teams also attended a conditioning clinic at the YMCA, and 10K/half participants were required to attend at least 2 practice runs in the community. This attendance allowed coaches to assess their students’ pace and endurance in addition to teaching students race etiquette.

As part of the program design, all students who completed 20 training miles would earn a pair of good-quality running shoes; however, in practice, nearly all participants received running shoes. Students who remained in the program until race day received free race registration, T-shirts, and an official medal. Coaches received a small stipend ($100–$300) at the conclusion of the program. Program size and incentives varied from year to year, depending on the level of external funding secured by the program.

### Study protocol

The evaluation team consisted of trained research staff of the Prevention Research Center for Healthy Neighborhoods and community partners, including school nurses, nursing and nutrition students, and YMCA fitness staff. Team members were trained and observed in their assigned protocol. The assessments took place at the YMCA 1 to 2 weeks before the beginning of training and again 1 to 2 weeks following race day.

### Measures

Participant age, sex, and race/ethnicity were self-reported. Weight and height were measured with the participant wearing light clothing and no shoes. Weight was measured to the nearest 0.1 kg using research-grade, calibrated, digital scales (Seca, model 882). Height was measured to the nearest 0.1 cm by using a free-standing portable stadiometer (Seca, model 213). Weight and height were measured twice, and the average of the 2 measurements was used to calculate BMI. We calculated BMI as weight in kilograms divided by the square of the height in meters (kg/m^2^). BMI percentile was calculated by using BMI-for-age growth charts, which are different for boys and girls, and then categorized at baseline as normal weight (<85th percentile), overweight (85th–94th percentile), or obese (≥95th percentile) (18).

We calculated waist-to-hip ratio (WHR) as the average waist measurement (cm) divided by the average hip measurement (cm). Higher ratios are an indicator of visceral fat around the abdomen, which is associated with cardiovascular disease, hypertension, and diabetes (19,20). Measurements were taken at the narrowest part of the waist and widest part of the hip, measured twice to the nearest 0.1 cm using a Gulick II tape measure (model 67020, North Coast Medical Inc) (21).

By using a nationally recommended protocol (22) and DuraShock cuffs (Welch Allyn, Inc), blood pressure readings were taken by experienced school nurses trained in the protocol. Participants were required to sit quietly for 5 minutes before the first measurement was taken. Seated, resting blood pressure and pulse were measured twice at each evaluation assessment. Measurements were taken using the right arm. If readings were substantially different (>10 mm Hg) or if 1 or more of the readings met the criteria for elevated blood pressure, a third reading was taken. The average of the readings was used in the analyses. Elevated blood pressure was defined as having a systolic and/or diastolic blood pressure at or above the 90th percentile for sex, age, and height (22). Students with elevated blood pressure readings were referred to the school nurse, who followed up with parents or guardians. Students with blood pressure at or above the 95th percentile were required to obtain a written release from their physician to continue with the program.

We used the sit-to-stand test to assess lower-extremity endurance and strength (23). Sitting in a chair approximately 17 inches from the ground (regardless of the height of the participant), students were asked to go from a sitting to a standing position and back to a sitting position 10 times as quickly and safely as possible. We used a stopwatch to record the time it took to complete all 10 repetitions to the nearest 0.01 second.

We used the Progressive Aerobic Cardiovascular Endurance Run (PACER) test, a multistage fitness test adapted from the 20-meter shuttle run test (24), to evaluate overall fitness. Following the published protocol, each participant runs 20-meter laps as the test gets progressively more difficult while an observer counts the number of laps completed (25).

Training dosage was measured as the total distance walked, jogged, or run during the 12- to 14-week training period. Before the program, routes within and outside the school grounds were measured by the coach and YMCA staff. Coaches documented distance completed, measured in tenths of a mile, after each training session, and they calculated accumulated mileage.

### Statistical analysis

We analyzed the data by using SPSS version 19 (IBM Corp). For elevated blood pressure, we examined proportional differences by using the McNemar test; for all other outcomes, repeated measures analysis of covariance (ANCOVA) were conducted with age, sex, and race/ethnicity as covariates. Both within-subject effects (change across time) and between-subject differences based on event (1.2 miles or 10K/half), training dosage (number of miles), and baseline BMI category were examined. Significance was set at *P* < .05.

## Results

The average age of participants was 13 years. Approximately half were male, and the racial/ethnic distribution was largely minority with 66% black, 13% Hispanic/Latino, 14% white, and 7% other race (including biracial) (Table 1). At baseline, one-third of participants were considered overweight (18%) or obese (17%), and nearly 15% had elevated blood pressure. Most participants (78.5%) enrolled in the 1.2-mile event, and 21.5% enrolled in the 10K/half events. Students who trained for the 10K/half events were older, and more African American students than students of other races/ethnicities trained for the 10K/half events.

**Table 1:** Comparison of Participant Characteristics in We Run This City Youth Marathon Program, by Training Event^a^, Cleveland, Ohio, 2009–2012

Characteristic	Baseline			Followed Versus Lost to Follow-Up					
				Total		1.2 Miles		10K or Half Marathon	
	Total (n = 1,419)^a^	1.2 Miles (n = 1,114)	10K or Half Marathon (n = 305)	Lost to Follow-Up (n = 464)	Followed (n = 955)	Lost to Follow-Up (n = 369)	Followed (n = 745)	Lost to Follow-Up (n = 95)	Followed (n = 210)
**Age, mean (SD), y**	13.00 (1.08)	12.96 (1.08)	13.16 (1.08)^b^	13.20 (1.04)	12.91 (1.09)^b^	13.19 (1.02)	12.85 (1.09)^b^	13.18 (1.12)	13.15 (1.06)
**Sex, %**									
Male	54.1	53.1	57.9	53.3	54.5	53.1	53.0	54.3	59.5
Female	45.9	46.9	42.1	46.7	45.5	46.9	47.0	45.7	40.5
**Race/ethnicity, %**									
Black	66.1	63.7	75.3^c^	63.8	67.2	62.0	64.6	71.3	77.1
White	14.4	15.7	9.1	14.8	14.2	15.4	15.9	12.6	7.4
Hispanic/Latino	13.0	14.0	9.1	15.7	11.6	17.9	12.0	6.9	10.1
Other race (including biracial)	6.6	6.6	6.5	5.6	7.0	4.7	7.5	9.2	5.3
**Baseline BMI^d^, %**									
Normal weight	64.5	63.2	69.0	61.9	65.6^c^	61.7	64.0	62.8	71.4
Overweight	18.2	18.2	18.1	16.7	18.9	15.9	19.4	20.5	17.2
Obese	17.3	18.5	12.8	21.4	15.5	22.5	16.7	16.7	11.3
**Baseline blood pressure^e^, %**									
Normal	85.2	85.3	84.7	88.1	83.9	88.4	83.9	86.8	83.8
Elevated	14.8	14.7	15.3	11.9	16.1	11.6	16.1	13.2	16.2

Abbreviation: BMI, body mass index.

a Students trained to run or walk a segment (1.2 miles, 6.2 miles [10K], or 13.1 miles [half marathon]) of the Rite Aid Cleveland Marathon. Study sample consists of first-time participants in We Run This City enrolled on a school team that completed the program.

b *P* < .001, χ^2^ test.

c *P* < .05, χ^2^ test.

d Based on BMI-for-age growth charts. (https://www.cdc.gov/healthyweight/assessing/bmi/childrens_bmi/about_childrens_bmi.html): normal weight, <85th percentile; overweight, 85th to 94th percentile; obese, ≥95th percentile. BMI calculated as weight in kilograms divided by the square of height in meters (kg/m^2^).

e Elevated blood pressure defined as systolic blood pressure and/or diastolic blood pressure at or above the 90th percentile for sex, age, and height.

Of students assessed at baseline, 67.3% completed the postintervention assessment. Overall, followed students were younger (*P* < .001) and fewer were obese (*P* = .03) than those in the lost to follow-up group, but did not differ by sex or race/ethnicity. When stratified by training event, only age was significant (followed students were younger) within the 1.2-mile event; no differences were found within the 10K/half event group. Primary reasons for withdrawing from the program or not attending the evaluation assessments, as reported by the coaches, were academic concerns, behavioral issues on the team or in school, transportation problems, illness, or injury.

Approximately half of participants met the goal of training 25 miles before the race (Figure). Because of the higher training expectations for the 10K/half groups, twice as many of the participants in these combined groups logged more than 25 miles than did those in the 1.2-mile event group. Although the number was not significant (*P* = .051), obese participants logged fewer miles than did either overweight or normal-weight participants. However, 39% of obese participants logged more than 25 miles of training, compared with 49% of normal-weight participants and 48% of overweight participants. We saw no difference in training levels between male and female participants.

**Figure:**
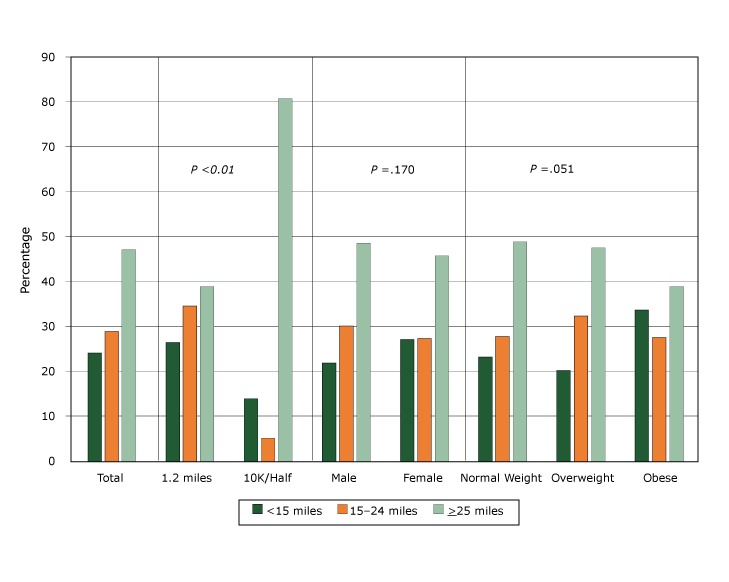
Total distance walked, jogged, or run (training dosage) by students followed over the 12- to 14-week training period for the We Run This City Youth Marathon Program, Cleveland, Ohio, 2009–2012. Students participated in a segment of 1.2 miles, 6.2 miles (10K), or 13.1 miles (half marathon). Body mass index (BMI) was based on BMI-for-age growth charts (normal weight, <85th percentile; overweight, 85th–94th percentile; obese, ≥95th percentile [https://www.cdc.gov/healthyweight/assessing/bmi/childrens_bmi/about_childrens_bmi.html]). **Table:** 

Category	Training Dosage			
	<15 miles	15–24 miles	≥25 miles	*P* Value
**Total**	24.1	28.8	47.1	Not applicable
**Event**				
1.2 miles	26.5	34.6	38.9	.001
10K/Half marathon	14.2	5.1	80.7	
**Sex**				
Male	21.7	30.1	48.3	.170
Female	27.0	27.3	45.7	
**BMI**				
Normal weight	23.2	27.7	49.0	.051
Overweight	20.1	32.3	47.6	
Obese	33.6	27.5	38.9

We observed significant improvements in both the sit-to-stand and the PACER test; on average, participants completed the sit-to-stand test 0.72 seconds faster (*P* = .02) and completed nearly 8 more laps in the PACER test (*P* < .001) than they did at baseline (Table 2). These significant effects held within the stratified models. Looking at the conditional effect of event, training dosage, or baseline BMI, we found only 1 significant interaction. While fitness improved in all 3 BMI groups, the change was markedly less among obese participants than among normal or overweight participants (*F* = 4.01; *P* = .02).

**Table 2:** Preintervention and Postintervention Differences in Participant (N = 955) Health and Fitness Measures, We Run This City Youth Marathon Program, Cleveland, Ohio, 2009–2012

Category	Preintervention, Adjusted Mean (SE)	Postintervention, Adjusted Mean (SE)	*F* a	*P *Value
**SIT-TO-STAND TEST ** **(** **23** **),** ** NO. OF SECONDS**				
**Adjusted for baseline age, sex, and race/ethnicity**				
**All participants**	9.54 (0.10)	8.82 (0.07)	5.18	.02
**By event**				
1.2 miles (n = 745)	9.48 (1.10)	8.74 (0.08)	Group, *F* = 3.17; *P* = .07 Time, *F* = 4.19; *P* = .04 Time × group, *F* = 0.13; *P* = .77	
10K or half marathon (n = 210)	9.77 (0.22)	9.11 (0.16)		
**By training dosage^b^**				
<15 miles	9.89 (0.20)	9.06 (0.14)	Group, *F* = 3.49; *P* = .03 Time, *F* = 5.02; *P* = .03 Time × group, *F* = 0.21; *P* = .81	
15–24 miles	9.30 (0.18)	8.61 (0.13)		
≥25 miles	9.46 (0.15)	8.79 (0.11)		
**By BMI^c^**				
Normal weight (n = 601)	9.41 (0.12)	8.61 (0.09)	Group, *F* = 7.03; *P* = .001 Time, *F* = 3.78; *P* = .05 Time × group, *F* = 0.88; *P* = .41	
Overweight (n = 173)	9.55 (0.23)	8.96 (0.16)		
Obese (n = 142)	10.06 (0.25)	9.52 (0.18)		
**By BMI, adjusted for age, sex, race/ethnicity, training dosage, and event**				
Normal weight (n = 601)	9.39 (0.12)	8.57 (0.09)	Group, *F* = 6.81; *P* = .001 Time, *F* = 3.72; *P* = .05 Time × group, *F* = 1.00; *P* = .37	
Overweight (n = 173)	9.60 (0.23)	8.98 (0.16)		
Obese (n = 142)	10.01 (0.26)	9.52 (0.19)		

**PACER TEST (** **24** **), NO. OF LAPS**				
**Adjusted for baseline age, sex, and race/ethnicity**				
**All participants**	28.13 (0.81)	35.71 (0.96)	14.57	<.001
**By event**				
1.2 miles (n = 745)	26.57 (0.96)	33.59 (1.04)	Group, *F* = 20.68; *P* < .001 Time, *F* = 17.2; *P* < .001 Time × group, *F* = 2.56; *P* = .11	
10K or half marathon (n = 210)	34.64 (1.96)	44.61 (2.14)		
**By training dosage^b^**				
<15 miles	24.16 (1.95)	30.12 (2.16)	Group, *F* = 4.52; *P* = .001 Time, *F* = 13.94; *P* < .001 Time × group, *F* = 1.10; *P* = .33	
15–24 miles	30.71 (1.57)	38.06 (1.73)		
≥25 miles	27.88 (1.21)	36.55 (1.34)		
**By BMI^c^**				
Normal weight (n = 601)	29.86 (1.07)	38.63 (1.56)	Group, *F* = 16.42; *P* < .001 Time, *F* = 11.74; *P* = .001 Time × group, *F* = 4.01; *P* = .02	
Overweight (n = 173)	28.57 (1.85)	36.44 (2.01)		
Obese (n = 142)	19.31 (2.21)	22.34 (2.39)		
**By BMI, adjusted for age, sex, race/ethnicity, training dosage, and event**				
Normal weight (n = 601)	29.42 (1.04)	38.47 (1.12)	Group, *F* = 20.10; *P* < .001 Time, *F* = 2.92; *P* = .09 Time × group, *F* = 3.74; *P* = .02	
Overweight (n = 173)	28.67 (1.80)	36.50 (1.94)		
Obese (n = 142)	19.76 (2.16)	23.03 (2.32)		

**BMI PERCENTILE**				
**Adjusted for baseline age, sex, and race/ethnicity**				
**All participants**	64.97 (0.97)	66.07 (0.92)	0.64	.43
**By event**				
1.2 miles (n = 745)	65.37 (1.09)	66.64 (1.04)	Group, *F* = 0.92; *P* = .33 Time, *F* = 0.14; *P* = .71 Time × group, *F* = 1.51; *P* = .22	
10K or half marathon (n = 210)	63.49 (2.11)	63.95 (2.01)		
**By training dosage^b^**				
<15 miles	68.69 (2.00)	68.96 (1.90)	Group, *F* = 2.73; *P* = .06 Time, *F* = 1.97; *P* = .66 Time × group, *F* = 4.18; *P* = .02	
15–24 miles	65.48 (1.84)	66.03 (1.74)		
≥25 miles	62.31 (1.42)	64.31 (1.35)		
**By BMI^c^**				
Normal weight (n = 601)	50.37 (0.81)	52.68 (0.80)	Group, *F* = 469.74; *P* < .001 Time, *F* = 0.39; *P* = .53 Time × group, *F* = 21.13; *P* <.001	
Overweight (n = 173)	90.16 (1.52)	88.97 (1.49)		
Obese (n = 142)	97.91 (1.72)	96.38 (1.69)		
**By BMI, adjusted for age, sex, race/ethnicity, training dosage, and event**				
Normal weight (n = 601)	50.48 (0.81)	52.88 (0.81)	Group, *F* = 438.48; *P* < .001 Time, *F* = 1.92; *P* = .17 Time × group, *F* = 21.76; *P* < .001	
Overweight (n = 173)	90.15 (1.55)	88.95 (1.53)		
Obese (n = 142)	97.64 (1.79)	96.14 (1.77)		

**WAIST-TO-HIP RATIO**				
**Adjusted for baseline age, sex, and race/ethnicity**				
**All participants**	0.80 (0.002)	0.81 (0.002)	0.01	.99
**By event**				
1.2 miles (n = 745)	0.80 (0.002)	0.81 (0.003)	Group, *F* = 1.96; *P* = .16 Time, *F* = 0.00; *P* = .99 Time × group, *F* = 0.00; *P* = .97	
10K or half marathon (n = 210)	0.73 (0.004)	0.80 (0.005)		
**By training dosage^b^**				
<15 miles	0.80 (0.004)	0.81 (0.005)	Group, *F* = 2.39; *P* = .09 Time, *F* = 0.10; *P* = .92 Time × group, *F* = 0.24; *P* = .80	
15–24 miles	0.79 (0.004)	0.80 (0.004)		
≥25 miles	0.80 (0.003)	0.81 (0.003)		
**By BMI^c^**				
Normal weight (n = 601)	0.79 (0.002)	0.79 (0.003)	Group, *F* = 73.26; *P* < .001 Time, *F* = 0.24; *P* = .63 Time × group, *F* = 3.41; *P* = .03	
Overweight (n = 173)	0.80 (0.005)	0.81 (0.005)		
Obese (n = 142)	0.84 (0.005)	0.86 (0.006)		
**By BMI, adjusted for age, sex, race/ethnicity, training dosage, and event**				
Normal weight (n = 601)	0.79 (0.002)	0.79 (0.003)	Group, *F* = 63.31; *P* < .001 Time, *F* = 0.25; *P* = .61 Time × group, *F* = 3.25; *P* = .04	
Overweight (n = 173)	0.80 (0.005)	0.81 (0.005)		
Obese (n = 142)	0.84 (0.005)	0.86 (0.006)		

Abbreviations: BMI, body mass index; PACER, Progressive Aerobic Cardiovascular Endurance Run test; SE, standard error.

a Pretest–posttest difference in outcomes tested by using repeated measures analysis of covariance (ANCOVA), controlling for age, sex, and race/ethnicity.

b Measured as the total distance walked, jogged, or run during the 12- to 14-week training period.

c Based on BMI-for-age growth charts. (https://www.cdc.gov/healthyweight/assessing/bmi/childrens_bmi/about_childrens_bmi.html): normal weight, <85th percentile; overweight, 85th to 94th percentile; obese, ≥95th percentile. BMI calculated as weight in kilograms divided by the square of height in meters (kg/m^2^).

Across the entire sample, we found no significant change in either BMI percentile or WHR. However, when the sample was stratified by BMI at baseline, different patterns emerged. For normal-weight participants, BMI percentile increased, whereas decreases in BMI percentile were observed for both overweight and obese participants (*F* = 21.13; *P* < .001). A significant interaction was also found for WHR, but in this case, a change was observed only in obese participants, and it was an increase in WHR rather than a decrease (*F* = 3.41; *P* = .03). In the final set of analyses for all 4 indicators (sit-to-stand test, PACER test, BMI, and WHR), the models were rerun, controlling for event and training dosage. The results (Table 2) did not change substantially.

In the nonstratified analyses, of the 137 participants with elevated blood pressure at baseline, 81.0% had readings in the normal range at postintervention, a substantial reduction even in light of the 6.8% of new cases of elevated blood pressure among the previously normal group (*P* < .001) (Table 3). This effect was largely sustained regardless of event, training dosage, or BMI at baseline; however, the subgroup analyses provide insight into the role of training intensity and BMI. For example, 93.5% of the 10K/half participants showed improvement compared with 77.4% of the 1.2-mile event participants (*P* < .001). Similarly, as the number of miles of training increased, the proportion with reductions in elevated blood pressure increased, from 72.7% for those with less than 15 miles of training to 77.8% for those with 15 to 24 miles and 85.7% for those with 25 or more miles of training. However, new cases of elevated blood pressure within the 15 to 24 miles group made the gains nonsignificant. With regard to BMI category, both normal weight and overweight participants showed significant improvements (82.1% and 89.7%, respectively). However, this was not the case for obese participants. Although 70% of those with elevated blood pressure at baseline had normal blood pressure postintervention, 30% continued to have elevated blood pressure and an additional 19 students (18.4% of those with normal blood pressure preintervention) had elevated blood pressure at postintervention. Moreover, even when participating at the highest training level (≥25 miles), only 73% of those with elevated blood pressure had normal blood pressure postintervention compared with 91% of overweight and 88% of normal weight peers. An additional 18% of these highly active but obese adolescents had elevated blood pressure after the intervention even though they had normal blood pressure at baseline.

**Table 3:** Changes in Blood Pressure Status^a^ Preintervention and Postintervention, Overall and Stratified by Event, Training Dosage, and BMI Category, We Run This City Youth Marathon Program, Cleveland, Ohio, 2009–2012

Postintervention Blood Pressure Category	Preintervention Blood Pressure Category		χ^2b^	*P*
	Normal, No. (%)	Elevated, No. (%)		
**Overall**	n = 721	n = 137	23.26	<.001
Normal	672 (93.2)	111 (81.0)		
Elevated	49 (6.8)	26 (19.0)		
**Event**				
**1.2 miles**	n = 567	n = 106	13.01	<.001
Normal	526 (92.8)	82 (77.4)		
Elevated	41 (7.2)	24 (22.6)		
**10K or half marathon**	n = 154	n = 31	10.81	.001
Normal	146 (94.8)	29 (93.5)		
Elevated	8 (5.2)	2 (6.5)		
**Training Dosage^c^**				
**<15 miles**	n = 161	n = 33	5.94	.02
Normal	152 (94.4)	24 (72.7)		
Elevated	9 (5.6)	9 (27.3)		
**15–24 miles**	n = 203	n = 36	3.35	.07
Normal	188 (92.6)	28 (77.8)		
Elevated	15 (7.4)	8 (22.2)		
**≥25**	n = 317	n = 63	10.78	.001
Normal	293 (92.4)	54 (85.7)		
Elevated	24 (7.6)	9 (14.3)		
**BMI Category^d^**				
**Normal weight**	n = 485	n = 78	19.55	<.001
Normal	463 (95.5)	64 (82.1)		
Elevated	22 (4.5)	14 (17.9)		
**Overweight**	n = 133	n = 29	8.50	.004
Normal	125 (94.0)	26 (89.7)		
Elevated	8 (6.0)	3 (10.3)		
**Obese**	n = 103	n = 30	0.03	.87
Normal	84 (81.6)	21 (70.0)		
Elevated	19 (18.4)	9 (30.0)		

Abbreviation: BMI, body mass index.

a Elevated blood pressure defined as systolic blood pressure and/or diastolic blood pressure at or above the 90th percentile for sex, age, and height.

b Proportional differences using the McNemar test.

c Measured as the total distance walked, jogged, or run during the 12- to 14-week training period.

d BMI was based on BMI-for-age growth charts (normal weight, <85th percentile; overweight, 85th–94th percentile; obese, ≥95th percentile [https://www.cdc.gov/healthyweight/assessing/bmi/childrens_bmi/about_childrens_bmi.html]).

## Discussion

Over the past decade, the amount of time during the school day dedicated to physical education has decreased dramatically. Simultaneously, the rate of obesity has grown exponentially. Both of these trends are markedly higher in economically disadvantaged communities and communities of color such as the one involved in this study. For these reasons, our study results provide promise. All participants, regardless of obesity status, appeared to have gained strength and endurance, as indicated in the 2 fitness measures. Overweight participants appeared to have benefited the most: increases in fitness, decreases in BMI, and improved heart health via normalized blood pressure, a result found by others (4). Obese participants, on the other hand, experienced a reduction in BMI and gained improved fitness, but at much lower levels than their peers, and far fewer experienced improvements in blood pressure, even when training at levels similar to normal weight peers.

Adolescents with obesity might find a school-based fitness program unwelcoming and daunting. However, when programs are developed to be inclusive and fun, we found that many overweight and obese students can and will stick to a moderate-to-vigorous physical activity program in their schools. The results also highlight the difficulty in retaining these most at-risk youth, as obese participants were more likely to drop out of the program compared with their peers.

This study also highlights the challenge of making a marked and sustained difference for adolescents who are already at the high end of the BMI charts. The increase in the WHR and the nonsignificant impact on blood pressure in this group suggests a complex physiologic interplay between physical activity, blood pressure, and visceral adiposity (19). Moderate levels of physical activity will improve blood pressure and visceral adiposity; however, the reviews are mixed, differing on the needed level of physical activity (4,9,26–28). Although many of the obese participants logged in as many miles as their peers, it is possible that the level of intensity was less (walking rather than running) and this explains the results.

We presume that part of the success of WRTC is due to the nature of a school–community partnership. In this case, the WRTC coaches are school personnel and the program is conducted during the school day, but tremendous support and resources are supplied by community partners. These partners not only support the school coaches but also provide the structure and financial backing for the program. As a result, the WRTC program appears to offer a unique experience: an opportunity that allows students to be physically active with friends and not just those in their gym class, perhaps allows them to interact with their teacher/coach in a different way than in a classroom setting, provides an opportunity to travel to different parts of the city for practice races and trainings, and has them run as a registered “runner” on race day with thousands of other runners, not to mention the experience of hearing thousands cheering as they cross the finish line. While unique, this program is one that should be replicable in other cities with a community race event and willing partners.

The lack of a comparison group as well as the cross-sectional study design are the primary limitations of this study. Without a comparison group, we cannot be certain that some of the improvements, particularly the fitness tests, were not due to a practice effect. Also, a longitudinal study of program participants would determine the effects of participation over several years and would strengthen support of this already successful program.

This study provides some evidence that the high rates of obesity could be reduced somewhat, or at least abated, if schools reinstated more frequent physical education programming during the school day, especially in districts where regular physical education has been drastically reduced over the past decade. One approach to bridging this gap is through strong, committed partnerships between schools and communities.
