# Erratum, Vol. 15, May 3 Release

**DOI:** 10.5888/pcd15.160471e

**Published:** 2018-05-17

Because of a technical error, an incorrect version of the article “We Run This City: Impact of a Community–School Fitness Program on Obesity, Health, and Fitness” appeared on our website. The PDF version of the article was correct as published.

Major corrections were to add the middle initial for author Meredith A. Goodwin and to correct the degree for Barbara A. Clint to BA. Other differences were several editorial changes to the article.

The article was corrected on May 4, 2018, and appears online at https://www.cdc.gov/pcd/issues/2018/16_0471.htm. We regret any confusion or inconvenience this error may have caused.

